# Supplementary material for: Enhancer histone-QTLs are enriched on autoimmune risk haplotypes and influence gene expression within chromatin networks
Source: Nat Commun. 2018 Jul 25;9:2905. doi: 10.1038/s41467-018-05328-9 (PMC6060153; doi:10.1038/s41467-018-05328-9)
Supplement: Supplementary file 1 — Supplementary Information [file 41467_2018_5328_MOESM1_ESM.pdf]

Supplementary Information

Enhancer histone-QTLs are enriched on autoimmune risk haplotypes and influence gene expression within chromatin networks

Pelikan et al.

Supplementary Figure 1. QQ plots of p-values from the CHT test for association between allele dosage and epigenetic read counts. The y-axis indicates the observed $-\log_{10}(p)$ versus expected $-\log_{10}(p)$ on the x-axis. Black points indicate statistics for the observed data and red points indicate statistics for the permuted data. The ordinal is drawn as a solid red line. H3K27ac is the top panel and H3K4me1 is the bottom panel.

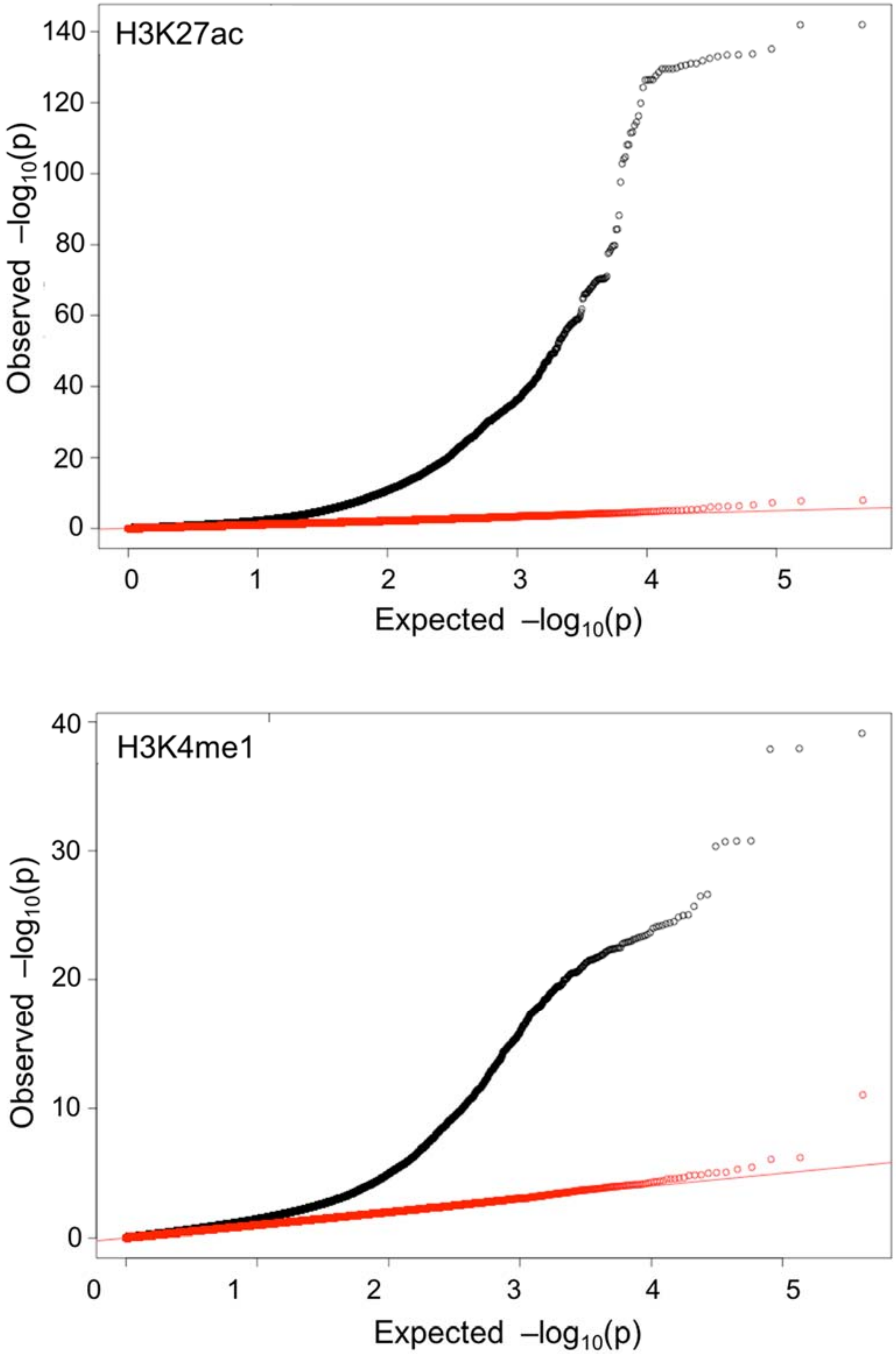

Supplementary Figure 2. H3K27a ChIP-seq read depth comparison between discovery (original) and replication samples. On average, the discovery cohort ChIP-seq data are, on average, 3.3 times deeper than the replication study ChIP-seq data. Box plots are presented by study (original 25 samples – blue; replication 10 samples – red) on the x-axis and sequencing reads (in millions) are plotted on the y-axis.

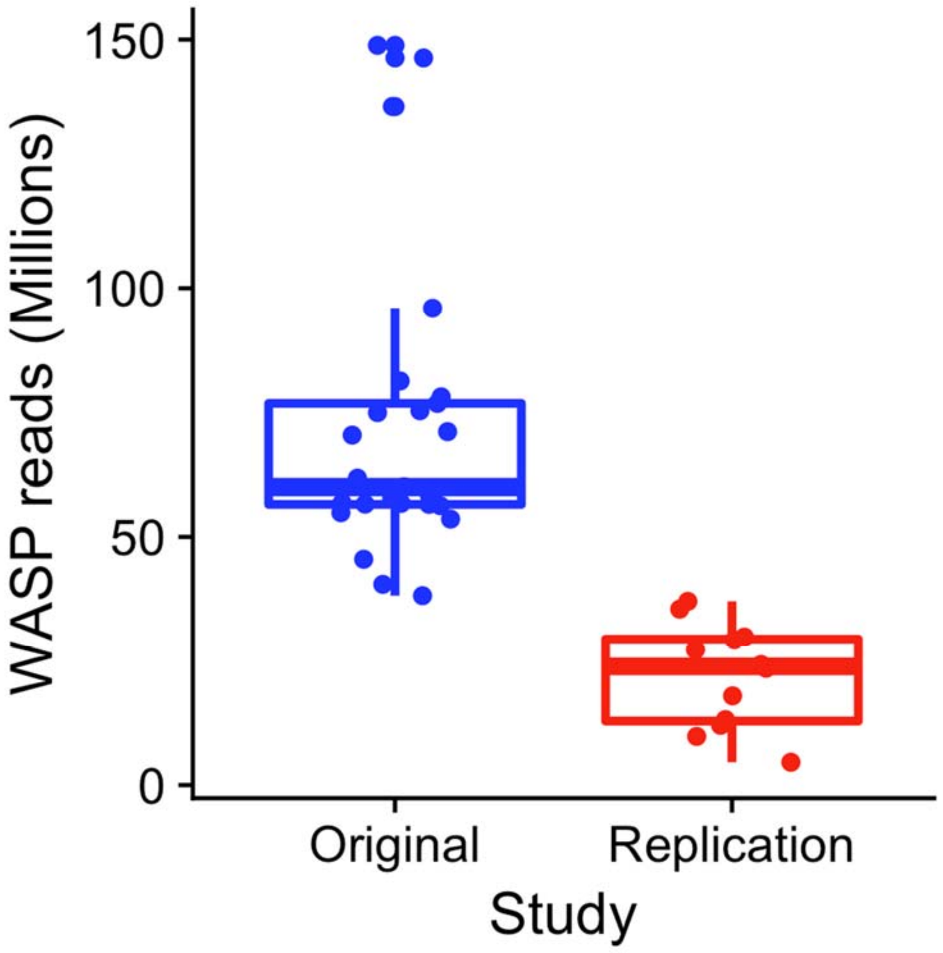

Supplementary Figure 3. Chromatin states and motif enrichment in H3K27ac and H3K4me1 consensus peaks. A. Predicted chromatin states of H3K27ac (black) and H3K4me1 (grey) consensus peaks. The majority of consensus peaks are primarily in enhancers, promoters, and transcriptional events. B. Motif enrichment of B cell lineage transcription factor binding sites within H3K27ac and H3K4me1 consensus peaks. Motif enrichment is plotted on the x-axis and the $-\log_{10}(p)$ on the y-axis.

A.

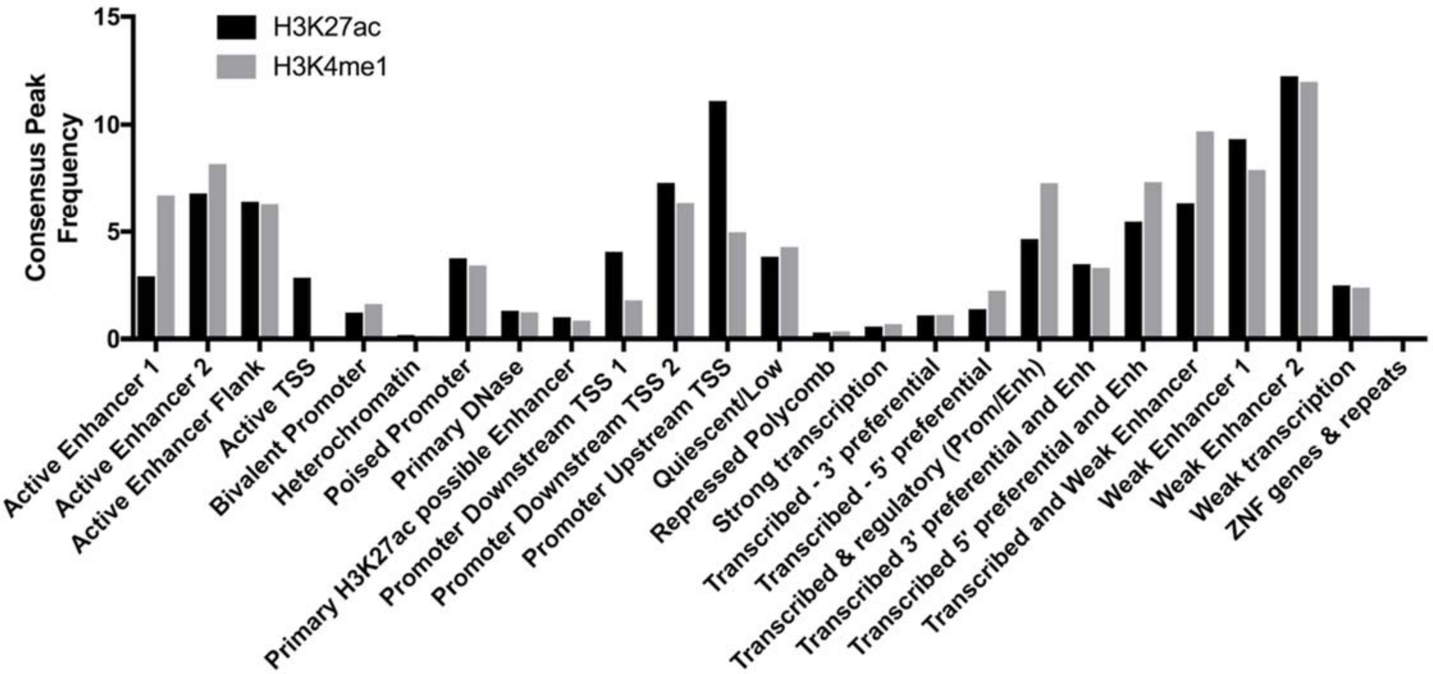

B.

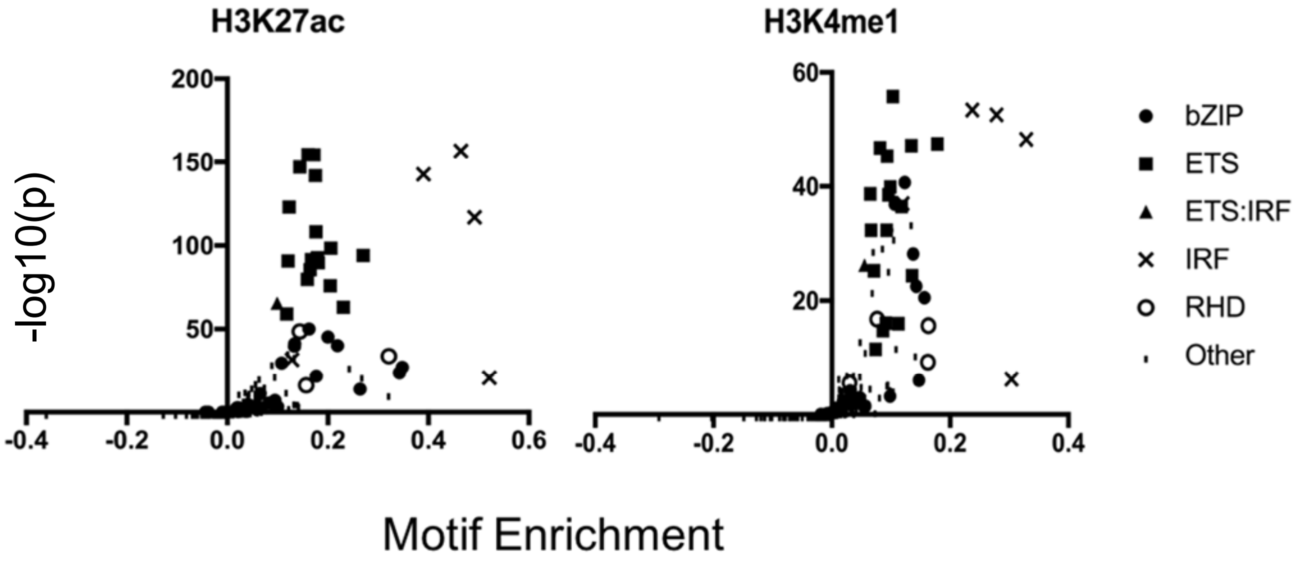

Supplementary Figure 4. Mirror plot of H3K27ac (top) and H3K4me1 (bottom) hQTL results for the HLA region on chromosome 6 (29.6 – 33.1 Mb). Y-axis is the $-\log_{10}(p)$ from the CHT; chromosome position is plotted on the x-axis and Class I, II, and III positions are indicated by colored bars. Red horizontal line indicates the 10% FEWR threshold; blue horizontal line indicates the 20% FEWR threshold.

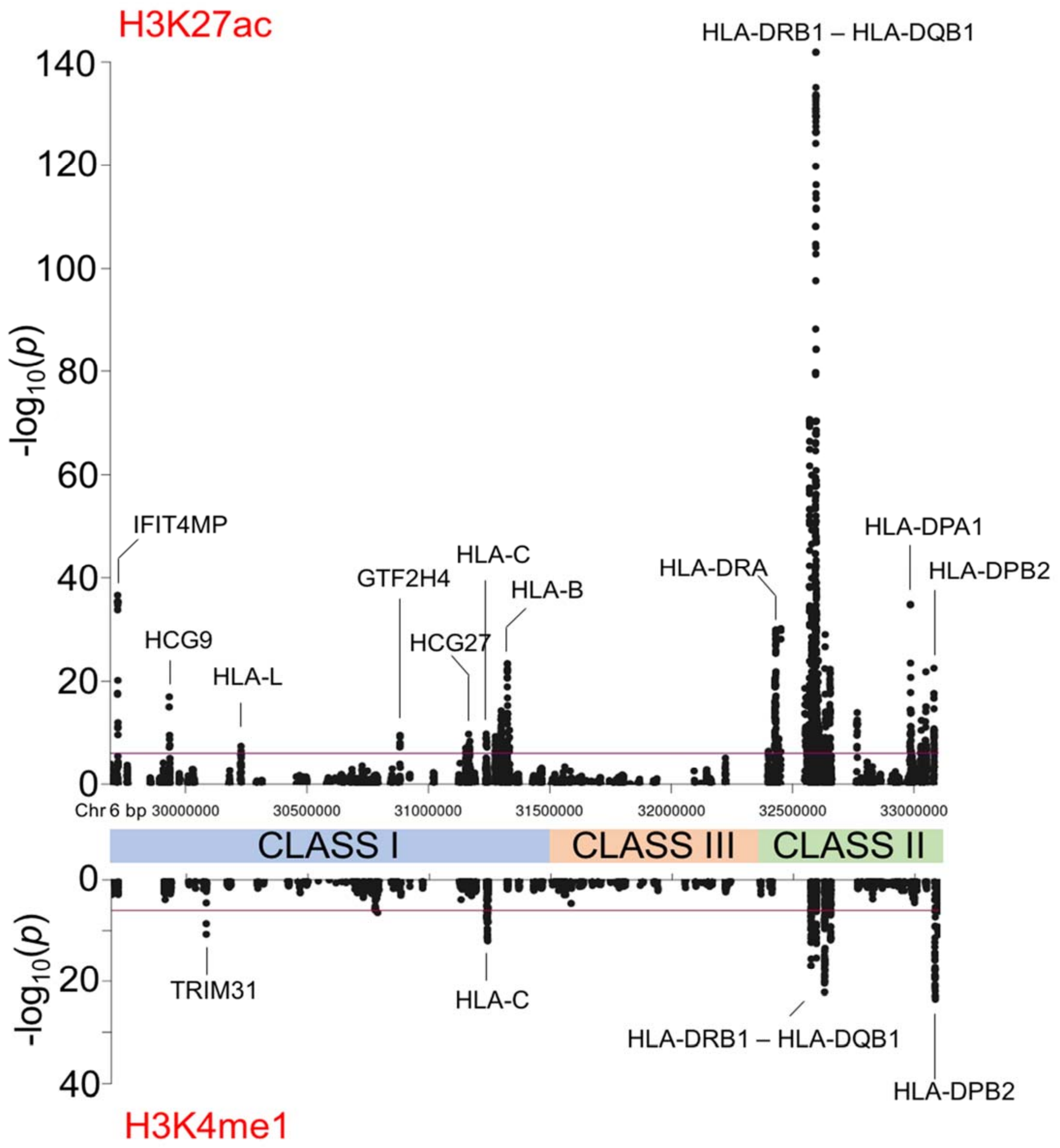

Supplementary Figure 5. Zoomed in HLA Class II region (32.54-32.64 Mb) showing haplotype-specific hQTLs separated by individuals heterozygous for the HLA-DR15 (top panel) or HLA-DR3 (bottom panel) haplotypes. hQTLs are colored by their r^2 with the peak hQTL in each region.

HLA-DR15

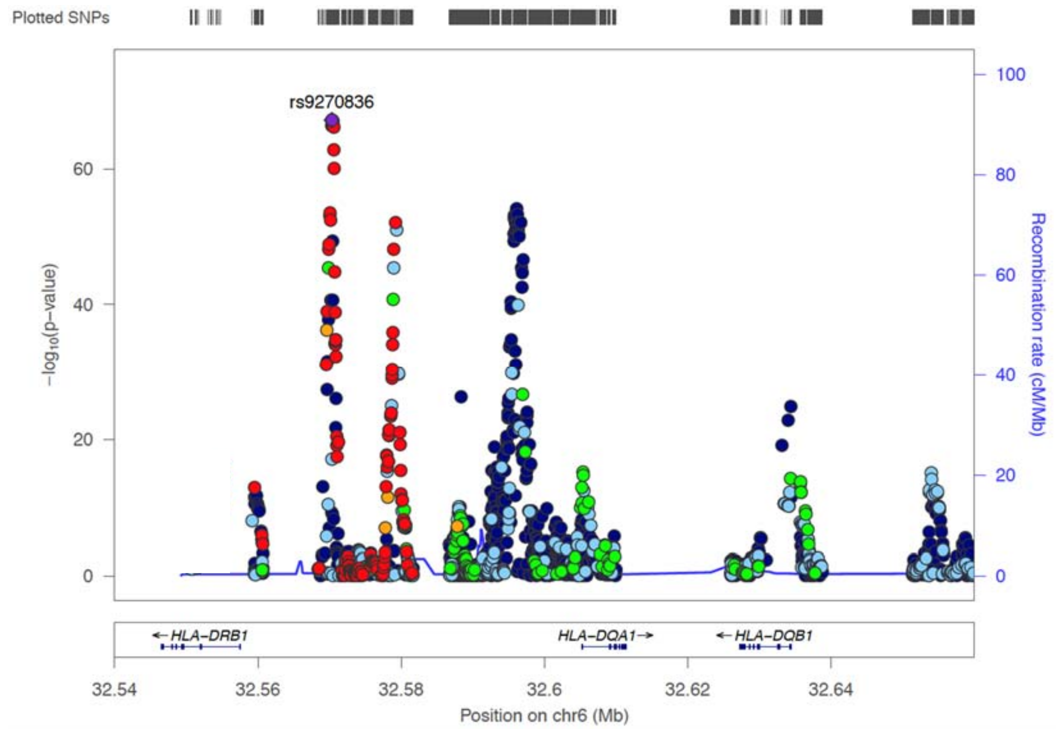

HLA-DR3

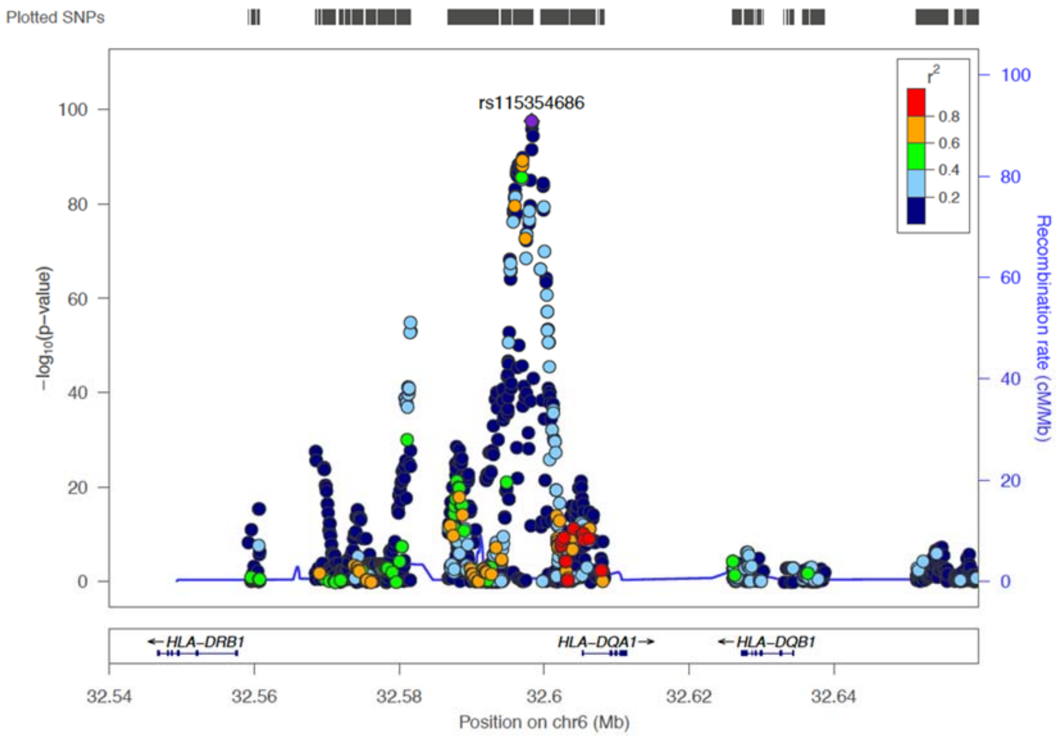

Supplementary Figure 6. HLA Class I hQTLs separated by individuals heterozygous for the HLA-DR15 (top panel) or HLA-DR3 (bottom panel) haplotypes. Y-axis is the -$\log_{10}$ (p) from the CHT; chromosome position (Mb) is plotted on the x-axis. hQTLs are colored by the $\log_2$ (DR15 allele specific reads / DR3 allele specific reads), with darker purple values (max = 3.68) indicating higher reads at a particular SNP in the HLA-DR15 samples compared to HLA-DR15 samples; stronger orange values (min = -3.04) indicating higher reads in the HLA-DR3 samples compared to HLA-DR15 samples at a particular SNP.

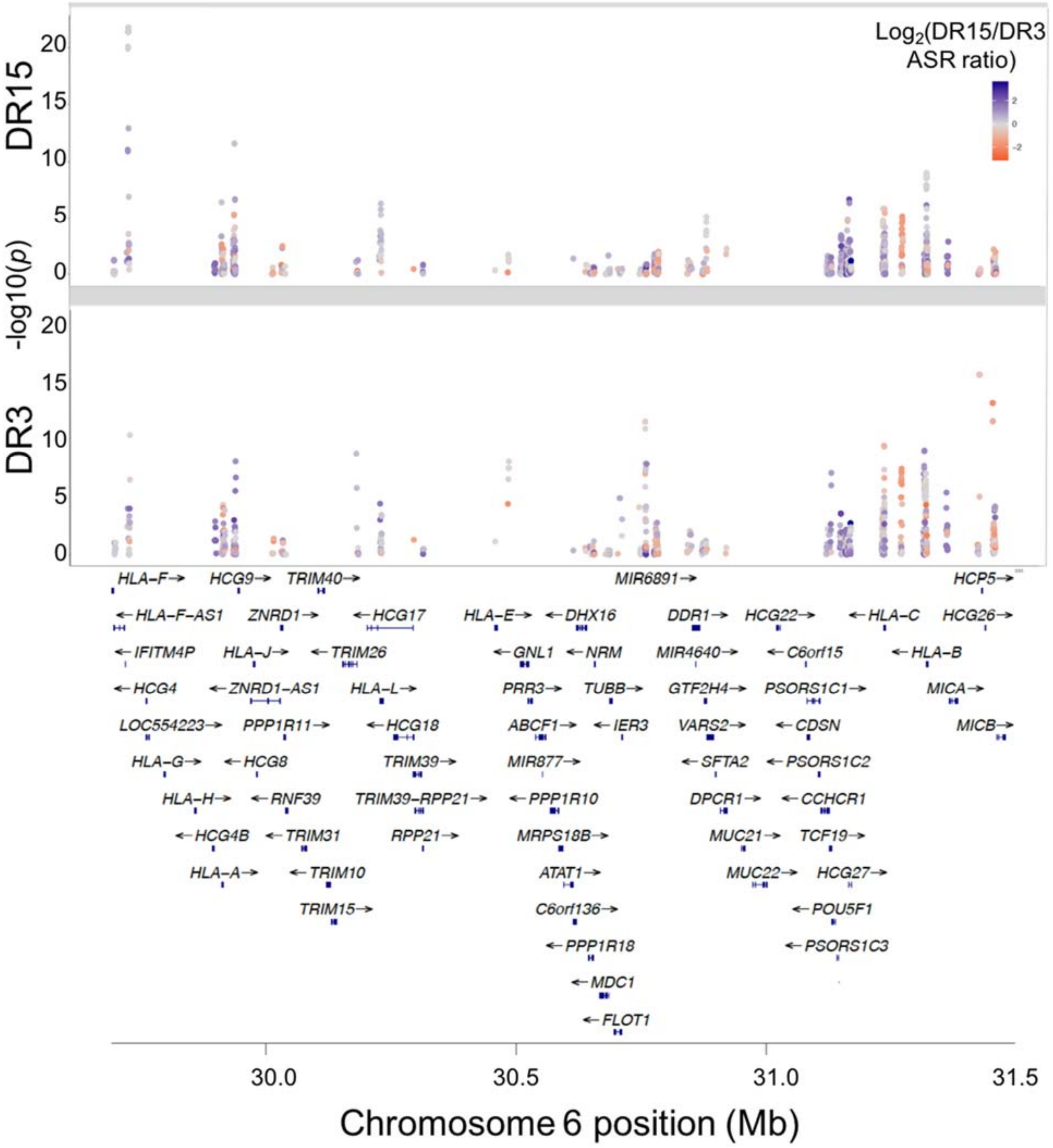

Supplementary Figure 7. HLA Class III hQTLs separated by individuals heterozygous for the HLA-DR15 (top panel) or HLA-DR3 (bottom panel) haplotypes. Y-axis is the $-\log_{10}(p)$ from the CHT; chromosome position (Mb) is plotted on the x-axis. hQTLs are colored by the $\log_2$ (DR15 allele specific reads / DR3 allele specific reads), with darker purple values (max = 3.25) indicating higher reads at a particular SNP in the HLA-DR15 samples compared to HLA-DR15 samples; stronger orange values (min = -4.62) indicating higher reads in the HLA-DR3 samples compared to HLA-DR15 samples at a particular SNP.

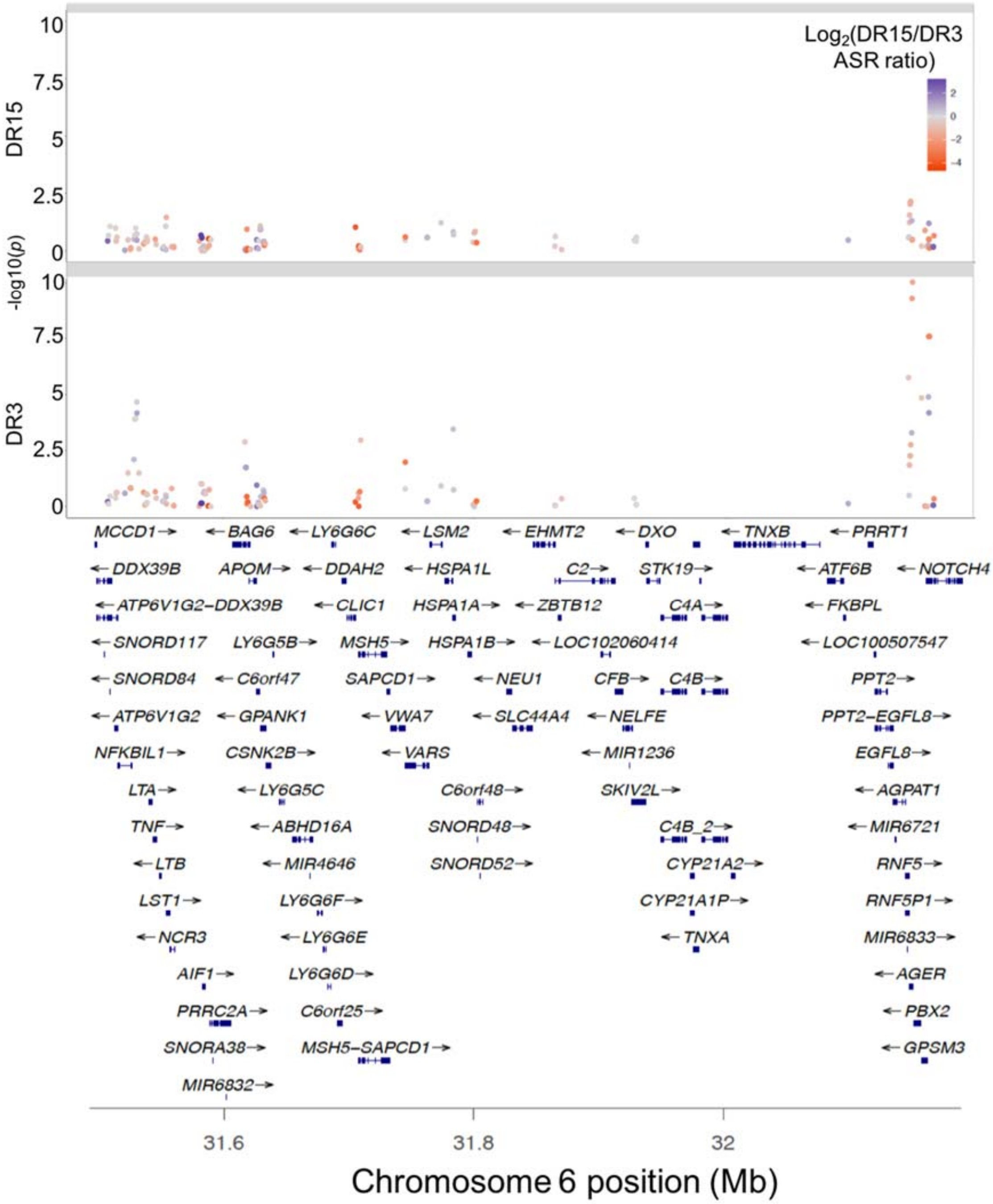

Supplementary Figure 8. CTCF Hi-ChIP and Hi-C data define insulated neighborhood around 32.5 Mb on chromosome 6 that includes HLA-DRA to HLA-DQB1. CTCF Hi-ChIP looping data are provided for a HLA-DR3 individual (top panel) and HLA-DR15 individual (middle panel). Heatmap of Hi-C data for GM12878 cell line is presented in bottom panel. CTCF data demonstrate similar looping and strength across the HLA-Class II region between the two HLA haplotypes except for in the region containing HLA-DRA to HLA-DQB1 (black box), which appears to be an insulated neighborhood as observed by the CTCF looping data and confirmed by TAD boundaries in the Hi-C data. Chromosome 6 positions (Mb) and important gene locations are provided along the bottom of the figure.

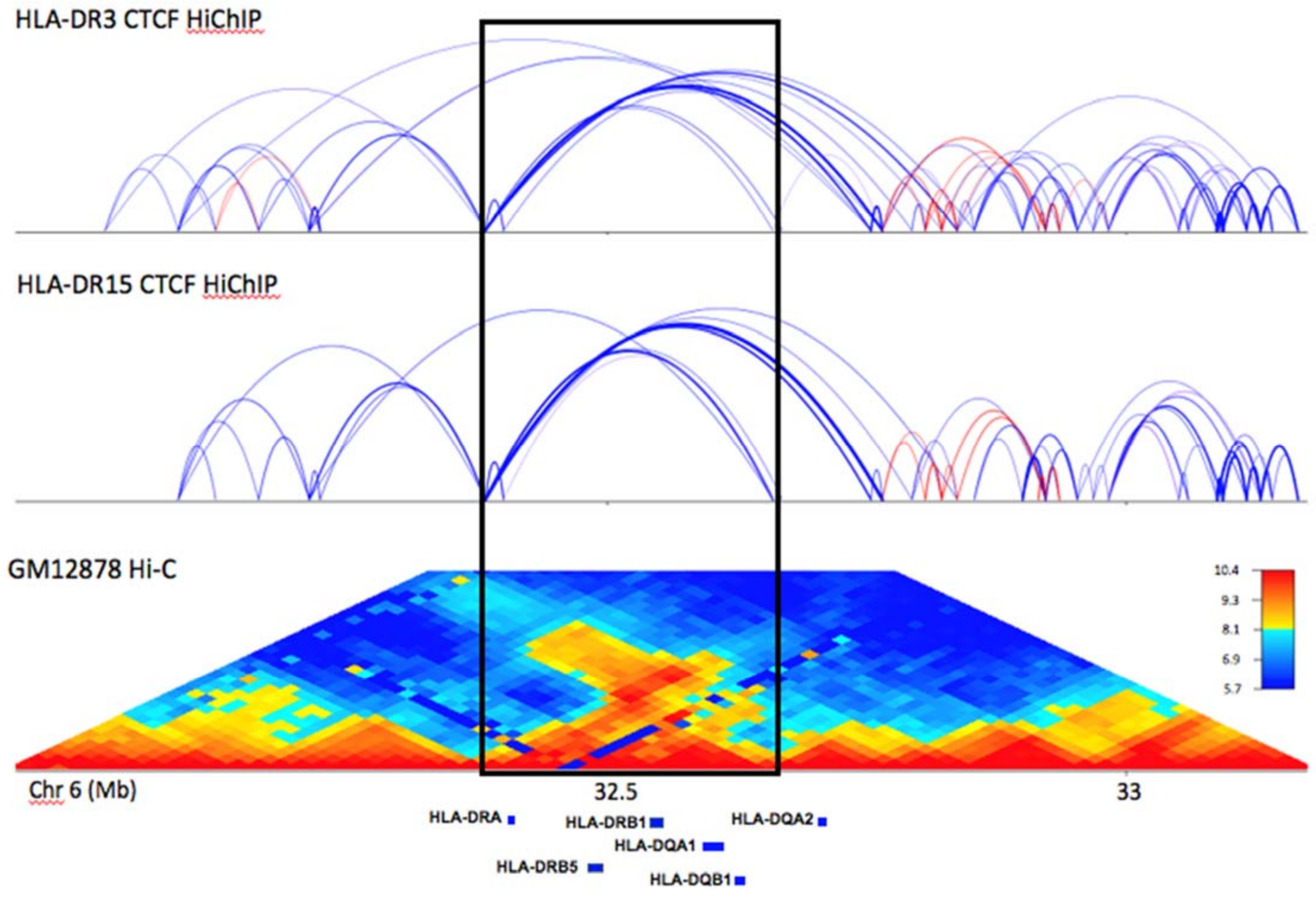

Supplementary Figure 9. Significant hQTL x eQTL distal interaction classification scheme. Spans of D' linkage disequilibrium (LD) are symbolized as hashed triangles. H3K27ac-mediated HiChIP interactions are depicted as dashed arcs and horizontal line segments for their anchor positions. The positions of hQTLs (solid dots) and eQTLs (circles) are indicated. The eQTL target gene is depicted as a bent arrow and exon-intron ideogram. The number and proportion of all significant distal interactions of each type are given. The majority of significant distal interactions are of the type where hQTLs interact distally with an eQTL target gene's chromatin network through chromatin looping. A. "Looped joint" interactions: the hQTL loops to the eQTL and eQTL target gene promoter, which are in LD. B. "Looped Disjoint" interactions: the hQTL loops directly to the eQTL target gene and the eQTL lies on a different haplotype block. C. "Shared anchor" interactions: LD-independent hQTL and eQTL are within the same loop anchor and interact with the eQTL target gene. D. "Shared anchor off-target" interactions: LD-independent hQTL and eQTL are within the same loop anchor and the significant interaction occurs with a gene that is not the eQTL target gene but is nevertheless within the 3D chromatin network of the hQTL. E. "Off-target" interactions: hQTL and eQTL are not within the same loop anchor and the significant interaction occurs with a gene that is not the eQTL target gene but is nevertheless within the 3D chromatin network of the hQTL. F. "Unanchored" interactions: the hQTL's haplotype block overlaps both a loop anchor and the eQTL target gene's regulatory area but is not explicitly covered by an interaction loop.

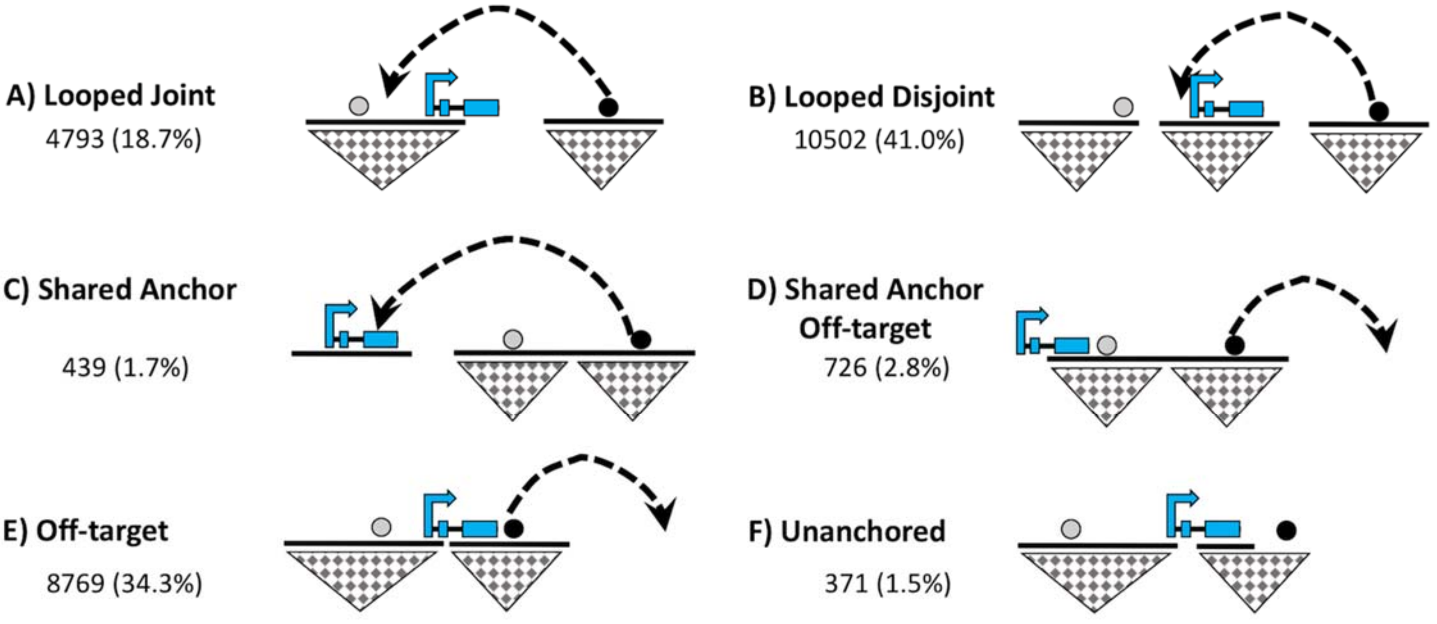

Supplementary Table 1. Distribution of p-values for 5,068 testable hQTLs in the 10-sample replication cohort.

Nominal p-value	# Replicated SNPs	% Replicated SNPs	Cumulative %age
< 0.000001	146	2.88	2.88
0.00001 > X > 0.000001	88	1.74	4.62
0.0001 > X > 0.00001	183	3.61	8.23
0.001 > X > 0.0001	344	6.79	15.02
0.01 > X > 0.001	621	12.25	27.27
0.05 > X > 0.01	799	15.77	43.03
NS	2887	56.97	100.0

Supplementary Table 2. hQTLs are located within the chromatin network of autoimmune disease risk genes.

**Autoimmune Disease**	**Risk Gene***
Ankylosing Spondylitis	B3GNT2; CARD9; HLA-B; RUNX3
Behcet's disease	HCG22; MICA; MICB
Celiac disease	CIITA; CLEC16A; ELMO1; HLA-DQA1; HLA-DQB1; ICOSLG; IRF4; ITGA4; KLK15; PFKFB3; PLEK; RGS1; RUNX3; SOCS1; TNFAIP3; UBASH3A; UBE2L3; YDJC
Crohn's disease	AIF1; AKAP1; ADCY3; CCR6; CD244; CD27; CNTNAP2; DCLRE1B; DNMT3A; ELF1; ERAP2; ESRRA; GALC; GLYAT; GPR35; GRP65; GPX4; GSDMB; HLA-DRA; HLA-DRB1; HLA-DQA1; ICOSLG; IFNGR2; IFNAR1; IFNAR2; IKZF3; IL2RA; INPP5D; IRF4; JAK2; KLF3; LACC1; LITAF; NOTCH4; ORMDL3; PBX2; PDCD1; PLCL1; PTK2B; PTPN22; RASGRP1; RBX1; REL; RNASET2; RUNX3; SLC25A28; SCL2A4RG; SLC43A3; SMNDC1; SP140; SPRED1; SYK; TBC1D1; TEF; THADA; TNFRSF1A; TFNSF8; TNFSF11; WBP4; YDJC; ZNF767P; ZFP91-CNTF; ZPBP2
Dermatomyositis	PLCL1
Graves' disease	C1QTNF6; HLA-A; HLA-B; HLA-G; RHOH; RNASET2
Kawasaki disease	BLK; FAM167A; FCGR2A; HLA-DOB; HLA-DQB2; PELI1
Multiple sclerosis	ALPK2; BATF; BOD1L2; BTNL2; CBLB; CD86; CHST12; CLEC16A; CLECL1; DIRAS2; DKKL1; EXTL2; FBXO48; GPR65; HLA-B; HLA-DQA1; HLA-DQB1; HLA-DRA; HLA-DRB1; HLA-DRB5; IL22RA2; IL2RA; IL7; ILDR1; IRF8; LINC-ROR; MAP3K14; MAPK1; MPHOSPH9; MYB; NFKBIZ; ODF3B; PLCL2; PLEK; RGS1; RREB1; SAE1; SCL30A7; SP140; SYK; TCF7; THADA; TNFRSF1A; VAV2; YWHAG; ZBTB46; ZNF767
Myesthenia gravis	HLA-DQA1; HLA-DRB1; PTPN22
Myositis	ATP6V1G2; DDX39B; HCP5; HLA-B; HLA-C; HLA-DPA1; HLA-DPB1; HLA-DQA1; HLA-DQB1; HLA-DRB1; MICB
Primary biliary cirrhosis	CCDC88B; CLEC16A; EXO3L4; GSDMB; HLA-DQB1; IKZF3; IRF8; NAB1; ORMDL3; PLCL2; RPS6KA4; SOCS1; SPIB; SYNGR1; TNFRSF1A
Psoriasis	AKAP13; B3GNT2; CLIC6; ELMO1; EXOC2; HLA-C; IFNAR1; IL13; IRF4; NFKBIA; NFKBIZ; PLCL2; PSMA6; REL; REV3L, RUNX1; TNFAIP3; TRAF3IP2
Rheumatoid arthritis	ABHD6; AIRE; APOM; B3GNT2; BATF; BLK; C1QBP; CCR6; CD83; CLNK; FAM167A; FCGR2A; GATA3; GCH1; GSDMB; HLA-DQA1; HLA-DRA; HLA-DRB1; HLA-DRB5; IFNGR2; IKZF3; IL2RA; IL2RB; IL6R; INPP5B; IRF4; IRF8; MED1; MTF1; N4BP1; NFKBIE; PLCL2; PLD4; PPIL4; PRKCH; PTPN22; PXK; RAD51B; RASGRP1; RCAN1; REL; RUNX1; SPRED2; SYNGR1; TEC; TNFAIP3; TPD52; TXNDC11; UBASH3A; UBE2L3; WDFY4; YDJC; XNF774; ZPBP2
Sarcoidosis	CCDC88B
Scleroderma (systemic sclerosis)	HLA-DPA1; HLA-DPB1; HLA-DQB1; HLA-DRA; HLA-DRB1; HLA-DQA1; IRF8; NOTCH4
Sjögren's syndrome	BLK; HLA-DPB1; HLA-DQA1; HLA-DRA; HLA-DRB1; TNFAIP3
Systemic lupus erythematosus	ABHD6; APOBEC4; BIN1; BLK; C2; CDKN1B; CIITA; CLEC16A; CREBL2; CSNK2B; DRAB1; EDEM3; ELF1; FAM167A; FAM98B; FCGR2A; GATA3; GPR19; GRP2; HCG27; HLA-A; HLA-B; HLA-DQA1; HLA-DQB1; HLA-DRA; HLA-DRB1; IKZF1; IKZF2; IKZF3; IRF7; IRF8; KDM4C; LAMC1; LCP1; LINC00271; LOC105369519; LYST; MED1; MSH5; NCF2; NOTCH4; OVOL1; PHLDB1-CXCR5; PLD2; PTPN22; PXK; RABGAP1L; RAD51B; RASSF2; SLC15A4; SMG7; SOCS1; SPRED2; TCF7; TFNAIP3; UBE2L3; UHRF1BP1; WDFY4; YDJC; ZNF184
Type I diabetes	C1QTNF6; CD69; CLEC16A; CTSH; GSDMB; HLA-DRA; HLA-DRB1; IL2RA; LMO7; ORMDL3; PGM1; PHTF1; PTPN22; RASGRP1; UBASH3A; ZPBP2
Ulcerative colitis	ADCY3; AHR; CALM3; CARD9; CDH1; CEP72; CIITA; CNTNAP2; DAP; EXOC3; FCGR2A; GLYAT; GNA12; GPR35; GSDMB; HCG9; HDAC9; HLA-A; HLA-DQA1; HLA-DQB1; HLA-DRA; HLA-DRB1; HLA-DRB5; HLA-G; ICOSLG; IER3; IKZF3; IL17REL; IRF8; JAK2; LITAF; LSP1; NFKBIZ; NOTCH4; PTGIR; SLC9A3; TNFRSF6B; TNFSF8; ZFP90; ZFP91-CNTF; ZPBP2
Vitiligo	C1QTNF6; CASP7; CCR6; CLNK; HLA-A; HLA-B; HLA-C; HLA-DQA1; HLA-DRA; IL2RA; PTPN22; RBM17; RERE; RNASET2; TOB2; UBASH3A
Wegener's disease	DCTD; HLA-DPB1; SEMA6A

*Listed autoimmune disease risk genes that either 1) contain hQTLs within reported risk haplotypes (red) or 2) interact with hQTLs through chromatin looping events (black). Index SNP and risk gene data were downloaded from the NHGRI-EPI catalog of Published Genome-wide Association Studies database October 17, 2016. Risk haplotypes were constructed around the reported index SNP based on linkage disequilibrium (D'>0.8) via haploview.

Supplementary Table 3. H3K27ac ChIP-sequencing statistics from our population of 25 LCLs.

ID	Histone mark	Input reads	Trimmed reads	Decontaminated reads	Initial total mapped reads	Initial unique mapped reads	Remappable reads	Reference overlapping reads	Alternate overlapping reads	Successfully mapped reads
1	H3K27ac	126726111	121981105	119156192	108691418	18742607	11894873	10014628	3107708	11510397
2	H3K27ac	51894146	51805454	88170385	81218341	13598448	9072516	7647793	2357318	8783037
3	H3K27ac	97066197	95476618	93186936	86292585	13704622	9554843	8097934	2457578	9237044
4	H3K27ac	70080544	67881775	66077601	59046449	11081976	6570357	5507262	1715440	6352214
5	H3K27ac	86587577	85116332	83031516	74592377	13708569	8309383	6987059	2168066	8033338
6	H3K27ac	104588193	102903830	100669400	92995385	15068932	10268693	8642122	2658122	9961928
7	H3K27ac	63956631	63648163	67985551	62732176	11428475	6900947	5828758	1787737	6661867
8	H3K27ac	92461013	90405720	87988934	79220355	14430096	8826382	7423588	2304227	8529159
9	H3K27ac	74946927	74141316	72355318	66758626	11903217	7308099	6155449	1893137	7068724
10	H3K27ac	72000836	71019464	69353982	63908674	11253059	6931168	5865412	1778565	6704258
11	H3K27ac	95768531	95402165	67131757	61942896	11264862	6713150	5681170	1725606	6482318
12	H3K27ac	85409933	84782315	82480340	74999197	13580940	8252778	6921872	2149973	7983985
13	H3K27ac	47940418	47035198	45798126	39652823	7421453	4553552	3834589	1193757	4386596
14	H3K27ac	68409834	67897122	70276100	64640548	11615691	6973662	5913810	1778999	6733550
15	H3K27ac	72998716	72244050	70545967	65027133	11730515	7087905	5967052	1826316	6858664
16	H3K27ac	98391992	95841314	93506778	86304667	13871447	9573543	8089248	2470434	9253484
17	H3K27ac	50131921	49605217	48395409	42046194	7785395	4794640	4043029	1249044	4624352
18	H3K27ac	54544342	53616154	52351885	48322241	8974970	5330327	4481626	1386417	5155045
19	H3K27ac	57900423	57751651	67920884	62467950	11412611	6802306	5728880	1742547	6572776
20	H3K27ac	81530738	81501873	64388802	59485207	10555021	6475131	5454940	1683371	6259814
21	H3K27ac	199961706	191607671	187254435	171273995	29394978	19015220	16132005	4878349	18401178
22	H3K27ac	125572347	125432279	69876769	64423827	11535220	7001861	5903313	1806833	6772296
23	H3K27ac	103877045	103714066	70240415	65031462	11532288	7019326	5910975	1818132	6786974
24	H3K27ac	205047631	200681482	196331727	180253433	31341219	19681047	16693199	5063274	19033672
25	H3K27ac	181183405	177735499	173923461	159139267	26933314	17404162	14643229	4542410	16850486

The number of reads at each step of the data processing pipeline are given: the total # of sequences obtained from the sequencer (input reads), the # of reads surviving subsequent filtering steps via Trimmomatic (trimmed reads) and removal of contaminant reads (decontaminated reads), the # of unique filtered reads before alignment (unique filtered reads), the # of reads aligning to the human reference genome (initial total mapped reads), the # of uniquely mapped reads (initial unique mapped reads), the # of reads overlapping SNPs segregating in the LCL population (remappable reads), the # of remappable reads matching the reference and alternate allele (overlapping reads), and the # of overlapping reads which are successfully remapped to correct reference genome bias (successfully remapped reads).

Supplementary Table 4. H3K27ac ChIP-sequencing statistics from our population of 25 LCLs.

ID	Histone mark	Input reads	Trimmed reads	Decontaminated reads	Initial total mapped reads	Initial unique mapped reads	Remappable reads	Reference overlapping reads	Alternate overlapping reads	Successfully mapped reads
1	H3K4me1	157513130	155477832	150870705	138593598	23750223	15071456	12604563	3902703	14559231
2	H3K4me1	115330642	112533924	119769271	110411539	17911514	12118390	10141354	3140816	11697184
3	H3K4me1	123904541	122236447	119005013	109144055	17949885	11981690	10031101	3107696	11559282
4	H3K4me1	154434981	150041779	145619985	130778606	22312108	14682830	12331693	3810070	14169701
5	H3K4me1	154695507	152880185	148423830	134746760	22764857	15026740	12611381	3896829	14521604
6	H3K4me1	109521443	105490931	102352778	94052852	15430984	10234242	8553080	2645458	9877405
7	H3K4me1	102264676	100705619	143675958	127620389	23551397	14655396	12272751	3835984	14111584
8	H3K4me1	181476599	178608189	173184649	156435071	26114025	17554907	14741009	4557556	16944585
9	H3K4me1	185190699	184422793	179526142	159229897	29314876	17952282	14994302	4741335	17280146
10	H3K4me1	196100381	195178275	190029013	168122903	30916952	18942040	15843418	4985723	18237363
11	H3K4me1	95746337	94512136	132632532	117869464	21820136	13466302	11257126	3537741	12969097
12	H3K4me1	128977400	128216612	124370950	112675473	19152103	12453549	10430946	3229599	12021036
13	H3K4me1	267196379	263103512	256640191	226121157	37426103	24997250	21025210	6466271	23960844
14	H3K4me1	69581933	68002996	147787508	131123671	24153699	14735646	12339166	3857721	14179369
15	H3K4me1	143846967	143186280	139205232	123190500	22919597	13912044	11638355	3657432	13390270
16	H3K4me1	261128097	256618511	249335215	229470053	37149656	24999890	20875919	6503570	24155968
17	H3K4me1	51041606	50551699	49147652	43151692	7922417	4916658	4143360	1273619	4738862
18	H3K4me1	143058368	142380814	138692001	123296510	22848883	13893018	11607831	3661932	13381864
19	H3K4me1	119028150	115961661	150406123	133364251	24551574	15275651	12798576	3984130	14703582
20	H3K4me1	133120758	130524030	190331719	168752350	30652605	19026529	15905238	5009847	18318722
21	H3K4me1	145207294	143293335	138945610	127593183	22241421	13953620	11699592	3590952	13467522
22	H3K4me1	125263941	125171950	136088800	121111889	22505304	13659422	11437594	3584175	13145700
23	H3K4me1	99238833	98981480	143709970	127812687	23609339	14409348	12042545	3795719	13869048
24	H3K4me1	136129630	134599895	130588634	119850954	20807027	13089868	10981967	3364192	12641615
25	H3K4me1	179706519	177678923	172239239	159173942	27141696	17301384	14446733	4486567	16707777

The number of reads at each step of the data processing pipeline are given: the total # of sequences obtained from the sequencer (input reads), the # of reads surviving subsequent filtering steps via Trimmomatic (trimmed reads) and removal of contaminant reads (decontaminated reads), the # of unique filtered reads before alignment (unique filtered reads), the # of reads aligning to the human reference genome (initial total mapped reads), the # of uniquely mapped reads (initial unique mapped reads), the # of reads overlapping SNPs segregating in the LCL population (remappable reads), the # of remappable reads matching the reference and alternate allele (overlapping reads), and the # of overlapping reads which are successfully remapped to correct reference genome bias (successfully remapped reads).

Supplementary Table 5. H3K27ac and CTCF HiChIP-sequencing statistics for samples heterozygous for the HLA-DR3 (N=3) or HLA-DR15 (N=3) haplotypes.

		**H3K27ac HiChIP**		**CTCF HiChIP**	
**Sample number**	**HLA Haplotype**	**Total PETs**	**% long range interactions**	**Total PETs**	**% long range interactions**
1	HLA-DR3	231132809	23.51	92390968	21.34
2	HLA-DR3	41845040	23.37	50680134	20.35
3	HLA-DR3	39640737	21.96	39376017	19.81
4	HLA-DR15	65520972	27.15	107042962	22.08
5	HLA-DR15	35509764	23.19	35335748	21.22
6	HLA-DR15	52729360	21.22	53873305	17.01

PETs: Paired end tags; Samples had to have a minimum of 15% long interactions to pass stringent quality control.
